# A Manual of Clinical Medicine

**Published:** 1855-11

**Authors:** 


A Manual of Clinical Medicine and Physical Diagnosis. By T. H. TANNER, M. D., Licentiate of the Royal College of Physicians, &c., &c., to which is added the code of ethics of the American Medical Association; Philadelphia: Blanchard & Lea, 1855.

THIS little book is crowded to its utmost capacity with useful matter. Its object will be gathered from the preface.

"The following pages have been written with the intention of removing some of the difficulties which the student always—and the practitioner frequently—must encounter, while studying disease in its Protean forms at the bedside. Remembering my own impressions of bewilderment on beginning to "walk the hospital," I have honestly endeavored to simplify the task for others; and should this treatise be the means of doing so, I shall feel greatly rewarded for my exertions."

The work is especially designed for those just commencing practice, suggesting facts and principles already familiar, and thus aiding the memory.

We are glad to find that the publishers have bound with it the code of ethics of the American Medical Association.

